# Supplementary material for: The human touch: a meta-analysis of anthropogenic effects on plant-pollinator interaction networks
Source: PeerJ. 2024 Jun 27;12:e17647. doi: 10.7717/peerj.17647 (PMC11214738; doi:10.7717/peerj.17647)
Supplement: Supplemental Information 4 — 38 studies for the systematic review and 20 studies for the comprehensive meta-analyses. These datasets were employed to examine the potential influence of anthropization on the structural characteristics of plant-pollinator interaction networks (Table S1). Likewise, we presents the results of subgroup analyses, revealing significant variations in observed effects across different categories such as pollinator types, plant species, continents, anthropogenic activities, and climatic conditions. This detailed analysis provides a more refined understanding of the overall impact by acknowledging and exploring potential differences and variations within the included studies (Table S2). [file peerj-12-17647-s004.docx]

Supplemental file 2 for: **The human touch: a meta-analysis of anthropogenic effects on plant-pollinator interaction networks**

Table S1. The dataset comprised 38 studies for the systematic review and 20 studies for the complete set of meta-analyses. These were utilized to investigate the potential impact of anthropization on the structural patterns of plant-pollinator interaction networks.

| Metric | Taxonomic group | Climate | Anthropogenic activity | Trend | Reference |
| --- | --- | --- | --- | --- | --- |
| Nestedness | Bees | Af:Tropical rainforest climate | Agriculture | No effect | *Hagen & Kraemer, 2010* |
|  |  | Cfa: Humid subtropical climate | Fragmentation | Decrease | *Newton et al., 2018* |
|  |  | Cfa: Humid subtropical climate | Deforestation | Decrease | *Mathiasson & Rehan, 2020* |
|  |  | BSh: Hot semi-arid (steppe) climate | Fragmentation | Increase | *Ferreira et al., 2020* |
|  |  | Csa:Hot-summer Mediterranean climate | Urbanización | Decrease | *Traveset et al., 2018* |
|  |  | Csa:Hot-summer Mediterranean climate | Urbanización | Decrease | *Prendergast & Ollerton, 2021* |
|  |  | BWk:Cold desert climate | Intentional fires | No effect | *Peralta et al., 2017* |
|  | Bees and others | Csb: Warm-summer Mediterranean climate | Agriculture | No effect | *Morrison & Dirzo, 2020* |
|  |  | Aw:Tropical savanna, wet | Deforestation | No effect | *Escobedo-Kenefic et al., 2022* |
|  |  | Cfb:Marine west coast climate | Fragmentation | No effect | *Jauker et al., 2019* |
|  |  | Cfb:Marine west coast climate | Fragmentation | Decrease | *Grass et al., 2018* |
|  | Insects | Cfb:Marine west coast climate | Agriculture | No effect | *Motivans et al., 2021* |
|  |  | Cfb:Marine west coast climate | Agriculture | Decrease | *Vanbergen et al., 2017* |
|  |  | Csb: Warm-summer Mediterranean climate | Agriculture | No effect | *Olsson et al., 2021* |
|  |  | Cfa: Humid subtropical climate | Fragmentation | Decrease | *Burkle & Knight, 2012* |
|  |  | Cfa: Humid subtropical climate | Fragmentation | No effect | *Spiesman & Inouye et al., 2013* |
|  |  | Cfb:Marine west coast climate | Livestock | Decrease | *Vanbergen et al., 2014* |
|  |  | Aw:Tropical savanna, wet | Agriculture | Decrease | *Moreira, Boscolo & Viana, 2015* |
|  |  | Af:Tropical rainforest climate | Deforestation | No effect | *Adedoja & Kehinde, 2018* |
|  |  | Cfa: Humid subtropical climate | Fragmentation | Decrease | *Della Rocca et al., 2023* |
|  |  | BSk:Cold semi-arid (steppe) climate | Fragmentation | Decrease | *Santamaría et al., 2018* |
|  | Butterflies | Cfb:Marine west coast climate | Agriculture | No effect | *Banza, Belo & Evans, 2015* |
|  |  | Cfb:Marine west coast climate | Agriculture | Decrease | *Colom, Traveset & Stefanescu, 2021* |
|  | Hummingbirds | Aw:Tropical savanna, wet | Agriculture | Decrease | *Bustamante-Castillo, Hernández Baños & Arizmendi, 2020* |
|  |  | Aw:Tropical savanna, wet | Deforestation | Increase | *Díaz Infante, Lara & Arizmendi, 2020* |
|  | General | Aw:Tropical savanna, wet | Fragmentation | No effect | *Pinto et al., 2020* |
| H_2_’ | Bees | Af:Tropical rainforest climate | Agriculture | No effect | *Hagen & Kraemer, 2010* |
|  |  | Cfa: Humid subtropical climate | Fragmentation | Increase | *Newton et al., 2018* |
|  |  | BSh: Hot semi-arid (steppe) climate | Fragmentation | Decrease | *Ferreira et al., 2020* |
|  |  | Csa:Hot-summer Mediterranean climate | Urbanización | Increase | *Traveset et al., 2018* |
|  |  | BSk:Cold semi-arid (steppe) climate | Urbanización | Increase | *Mas & Vilagines, 2018* |
|  |  | Csa:Hot-summer Mediterranean climate | Urbanización | No effect | *Prendergast & Ollerton, 2021* |
|  |  | BWk:Cold desert climate | Intentional fires | Increase | *Peralta et al., 2017* |
|  | Bees and others | Cfb:Marine west coast climate | Agriculture | No effect | *Fründ, Linsenmair & Blüthgen, 2010* |
|  |  | Aw:Tropical savanna, wet | Deforestation | No effect | *Escobedo-Kenefic et al., 2022* |
|  |  | Cfb:Marine west coast climate | Fragmentation | Increase | *Jauker et al., 2019* |
|  | Insects | Cfa: Humid subtropical climate | Agriculture | No effect | *Weiner et al., 2011* |
|  |  | Cfb:Marine west coast climate | Agriculture | Increase | *Vanbergen et al., 2017* |
|  |  | Cfa: Humid subtropical climate | Agriculture | Increase | *Shinohara,Uchida & Yoshida, 2019* |
|  |  | Cfb:Marine west coast climate | Agriculture | Decrease | *Motivans et al., 2021* |
|  |  | Cfa: Humid subtropical climate | Fragmentation | Decrease | *Burkle & Knight, 2012* |
|  |  | Cfa: Humid subtropical climate | Fragmentation | Decrease | *Marrero, Torretta & Medan, 2014* |
|  |  | Cwb:Subtropical highland climate | Urbanización | Decrease | *Marín et al., 2020* |
|  |  | Af:Tropical rainforest climate | Deforestation | No effect | *Adedoja & Kehinde, 2018* |
|  |  | Am:Tropical monsoon climate | Deforestation | Increase | *Sritongchuay et al., 2022* |
|  |  | Cfa: Humid subtropical climate | Fragmentation | Decrease | *Della Rocca et al., 2023* |
|  |  | Cfa: Humid subtropical climate | Intentional fires | No effect | *Da Silva et al., 2022* |
|  |  | BSk:Cold semi-arid (steppe) climate | Fragmentation | Increase | *Santamaría et al., 2018* |
|  |  | Cfa: Humid subtropical climate | Agriculture | No effect | *Weiner et al., 2014* |
|  | Butterflies | Cfb:Marine west coast climate | Agriculture | Decrease | *Banza, Belo & Evans, 2015* |
|  |  | Cfb:Marine west coast climate | Agriculture | No effect | *Colom, Traveset & Stefanescu, 2021* |
|  | Hummingbirds | Aw:Tropical savanna, wet | Agriculture | No effect | *Bustamante-Castillo, Hernández Baños & Arizmendi, 2020* |
|  | General | Aw:Tropical savanna, wet | Fragmentation | No effect | *Pinto et al., 2020* |
| Conectance | Bees | Cfa: Humid subtropical climate | Fragmentation | Decrease | *Newton et al., 2018* |
|  |  | Cfa: Humid subtropical climate | Deforestation | Decrease | *Mathiasson & Rehan, 2020* |
|  |  | BSk:Cold semi-arid (steppe) climate | Urbanización | Decrease | *Mas & Vilagines, 2018* |
|  |  | Csa:Hot-summer Mediterranean climate | Urbanización | No effect | *Traveset et al., 2018* |
|  |  | Csa:Hot-summer Mediterranean climate | Urbanización | No effect | *Prendergast & Ollerton, 2021* |
|  |  | BWk:Cold desert climate | Intentional fires | Decrease | *Peralta et al., 2017* |
|  | Bees and others | Csb: Warm-summer Mediterranean climate | Agriculture | Increase | *Morrison & Dirzo, 2020* |
|  |  | Aw:Tropical savanna, wet | Deforestation | No effect | *Escobedo-Kenefic et al., 2022* |
|  |  | Cfb:Marine west coast climate | Fragmentation | Decrease | *Jauker et al., 2019* |
|  | Insects | Cfb:Marine west coast climate | Agriculture | Increase | *Vanbergen et al., 2014* |
|  |  | Cfa: Humid subtropical climate | Agriculture | Increase | *Shinohara,Uchida & Yoshida, 2019* |
|  |  | Cfb:Marine west coast climate | Agriculture | Decrease | *Motivans et al., 2021* |
|  |  | Csb: Warm-summer Mediterranean climate | Agriculture | No effect | *Olsson et al., 2021* |
|  |  | Cfa: Humid subtropical climate | Fragmentation | Increase | *Spiesman & Inouye et al., 2013* |
|  |  | Cfb:Marine west coast climate | Fragmentation | Decrease | *Librán‐Embid et al., 2021* |
|  |  | Cfb:Marine west coast climate | Livestock | Decrease | *Vanbergen et al., 2017* |
|  |  | Af:Tropical rainforest climate | Deforestation | No effect | *Adedoja & Kehinde, 2018* |
|  |  | Cfa: Humid subtropical climate | Fragmentation | Increase | *Della Rocca et al., 2023* |
|  |  | BSk:Cold semi-arid (steppe) climate | Fragmentation | Increase | *Santamaría et al., 2018* |
|  | Hummingbirds | Aw:Tropical savanna, wet | Agriculture | No effect | *Bustamante-Castillo, Hernández Baños & Arizmendi, 2020* |
|  |  | Aw:Tropical savanna, wet | Deforestation | No effect | *Díaz Infante, Lara & Arizmendi, 2020* |
|  | General | Af: Tropical rainforest climate | Agriculture | Increase | *Sritongchuay et al., 2019* |
| Modularity | Bees | BWk:Cold desert climate | Intentional fires | Increase | *Peralta et al., 2017* |
|  | Bees and others | Csb: Warm-summer Mediterranean climate | Agriculture | Increase | *Morrison & Dirzo, 2020* |
|  |  | Aw:Tropical savanna, wet | Deforestation | No effect | *Escobedo-Kenefic et al., 2022* |
|  |  | Cfb:Marine west coast climate | Fragmentation | Increase | *Grass et al., 2018* |
|  | Insects | Cfb:Marine west coast climate | Agriculture | Increase | *Villa‐Galavi et al., 2021* |
|  |  | Cfa: Humid subtropical climate | Fragmentation | Increase | *Spiesman & Inouye et al., 2013* |
|  |  | Cfb:Marine west coast climate | Fragmentation | Increase | *Librán‐Embid et al., 2021* |
|  |  | Cfa: Humid subtropical climate | Intentional fires | No effect | *Da Silva et al., 2022* |
|  |  | BSk:Cold semi-arid (steppe) climate | Fragmentation | Increase | *Santamaría et al., 2018* |
|  | Butterflies | Cfb:Marine west coast climate | Agriculture | Decrease | *Colom, Traveset & Stefanescu, 2021* |
|  | Hummingbirds | Aw:Tropical savanna, wet | Agriculture | No effect | *Bustamante-Castillo, Hernández Baños & Arizmendi, 2020* |

Bees, refers to studies considering only these hymenopteran species. Bees and others; refers to studies considering a group of pollinators consisting of species of bees, hoverflies, bumblebees, and wasps. Butterflies; refers to studies considering only lepidopteran species. Insects; refers to studies considering species from all the aforementioned groups as pollinators. Hummingbirds, refers to studies considering only this bird group as pollinators. General, refers to studies considering collectively mammals and species from all the aforementioned groups as pollinators.

Table S2. The subgroup analyses unveil notable differences in observed effects among subgroups of pollinators, plants, climate, anthropogenic activities, and continent. This detailed analysis offers a more nuanced understanding of the overall effect by accounting for potential differences and variations present across the included studies.

| Category | SMD  *size effect* | 95% confidence intervals | *P*  (within groups) | Tau^2^ |
| --- | --- | --- | --- | --- |
| **Species** |  |  | 0.02 |  |
| Pollinators | 0.79 | 0.22*–*1.36 |  | 0.88 |
| Plants | 0.13 | -0.58*–*0.85 |  | 1.37 |
| **Climate** |  |  | 0.0001 |  |
| Marine west coast climate | 0.99 | -0.24-2.23 |  | 0.56 |
| Humid subtropical climate | 0.09 | -0.44-0.64 |  | 0.56 |
| Subtropical highland climate | -0.89 | -14.2-12.41 |  | 0.56 |
| Tropical monsoon climate | 1.46 | -8.29-11.21 |  | 0.56 |
| Tropical rainforest climate | -0.25 | -17.88-17.38 |  | 0.56 |
| Warm-summer Mediterranean climate | -0.85 | -2.46-0.76 |  | 0.56 |
| Tropical savanna, wet | 1.85 | -8.48-12.2 |  | 0.56 |
| Hot semi-arid (steppe) climate | 2.04 | -0.86-4.95 |  | 0.56 |
| Cold desert climate | 0.34 | -1.3-1.98 |  | 0.56 |
| Hot-summer Mediterranean climate | 0.57 | 0.05-1.1 |  | 0.56 |
| **Anthropogenic activity** |  |  | 0.02 |  |
| Agriculture | 0.15 | -0.32*–*0.62 |  | 0.62 |
| Fragmentation | 2.07 | 1.06*–*3.09 |  | 0.11 |
| Urbanization | -0.44 | -4.54*–*2.76 |  | 0.82 |
| Deforestation | 0.98 | 0.11*–*1.85 |  | 0.36 |
| Fires | -0.49 | -2.57*–*1.72 |  | 2.26 |
| **Continent** |  |  | 0.02 |  |
| America | 0.66 | -0.55*–*1.87 |  | 2.64 |
| Europe | 0.50 | 0.003*–*1.01 |  | 0.57 |
| Africa | 0.50 | -0.78*–*1.80 |  | 0.84 |
| Asia | 0.50 | -2.33*–*2.93 |  | 1.59 |
