# Supplementary material for: The human touch: a meta-analysis of anthropogenic effects on plant-pollinator interaction networks
Source: PeerJ. 2024 Jun 27;12:e17647. doi: 10.7717/peerj.17647 (PMC11214738; doi:10.7717/peerj.17647)
Supplement: Supplemental Information 5 — The rationale for conducting a meta-analysis on the impact of anthropogenic activities on plant-pollinator interaction networks and situates the study within the context of existing literature. It discusses how the meta-analysis contributes to the current understanding of the topic by incorporating a wider range of factors, network metrics, and regional differences, while highlighting the need for further research in this area. [file peerj-12-17647-s005.docx]

Supplemental file 3

Rationale for conducting the meta-analysis:

The meta-analysis on the effects of anthropogenic activities on plant-pollinator interaction networks is crucial given the escalating human impact on natural ecosystems. As human populations continue to grow and expand, their influence on the environment becomes increasingly pronounced. Anthropogenic factors such as deforestation, urbanization, habitat fragmentation, agriculture, intentional fires, and livestock farming have the potential to significantly alter the composition and structure of plant and pollinator communities. These changes can have far-reaching consequences for pollination services, which are vital for the maintenance of biodiversity and the functioning of ecosystems.

Despite the growing body of literature on plant-pollinator networks, the responses of these networks to anthropogenic pressures remain poorly understood. Individual studies often focus on specific anthropogenic factors or geographic regions, making it challenging to derive generalizable conclusions. By conducting a meta-analysis, we aim to synthesize the available evidence and provide a comprehensive assessment of how various anthropogenic activities reshape the structure of plant-pollinator networks across different ecosystems and regions.

The meta-analysis allows us to quantitatively evaluate the effects of anthropization on key network metrics such as nestedness, specialization, connectance, and modularity. By examining these metrics, we can gain insights into how human activities alter the interactions between plants and pollinators, potentially leading to changes in network stability, resilience, and function. Furthermore, by incorporating data on species richness, we can assess the impact of anthropization on biodiversity within these networks.

The findings of this meta-analysis have important implications for conservation and management strategies. By identifying the anthropogenic factors that have the most significant impact on plant-pollinator networks, we can prioritize conservation efforts and develop targeted interventions to mitigate the negative effects of human activities. Moreover, understanding the regional differences in network responses can inform the development of context-specific management plans that account for the unique characteristics of different ecosystems.

Contribution to knowledge in light of previously published related reports:

Our meta-analysis on the effects of anthropogenic activities on plant-pollinator interaction networks builds upon and extends the existing knowledge in this research area. While previous meta-analyses and systematic reviews have explored the impact of human activities on pollination services and species richness, our study offers several unique contributions.

Firstly, our meta-analysis incorporates a wider range of anthropogenic factors compared to previous studies. By including deforestation, urbanization, habitat fragmentation, agriculture, intentional fires, and livestock farming, we provide a more comprehensive assessment of how different human activities shape plant-pollinator networks. This broader scope allows for a more nuanced understanding of the relative importance of each factor and their potential interactions.

Secondly, our study examines specific network metrics that have not been consistently analyzed in previous meta-analyses. By focusing on nestedness, specialization, connectance, and modularity, we offer new insights into how anthropogenic activities alter the structure and organization of plant-pollinator networks. These metrics provide valuable information about the stability, resilience, and functioning of these networks, which is crucial for predicting their responses to future environmental changes.

Thirdly, our meta-analysis reveals regional differences in network responses to anthropization. By considering studies from various geographic locations, we highlight the context-dependent nature of anthropogenic effects on plant-pollinator networks. This finding emphasizes the need for tailored conservation and management strategies that account for the unique characteristics of different ecosystems.

Furthermore, our study identifies habitat fragment size as a key variable contributing to the heterogeneity in species richness responses to anthropization. This insight underscores the importance of considering landscape-level factors when assessing the impact of human activities on biodiversity within plant-pollinator networks.

In comparison to previous meta-analyses and systematic reviews, our study provides a more comprehensive and nuanced understanding of how anthropogenic activities reshape plant-pollinator interaction networks. By incorporating a wider range of factors, examining specific network metrics, and considering regional differences, we contribute new knowledge that can inform conservation and management efforts.

However, it is important to acknowledge that our meta-analysis also highlights the need for further research in this area. While we provide valuable insights, the complex nature of plant-pollinator networks and the multitude of anthropogenic pressures they face require ongoing investigation. Future studies should focus on filling knowledge gaps, exploring the mechanisms underlying network responses, and developing predictive models to anticipate the consequences of human activities on these critical ecological interactions.
